# Younger age of stroke in low‐middle income countries is related to healthcare access and quality

**DOI:** 10.1002/acn3.51507

**Published:** 2022-02-09

**Authors:** Mohammad H. Rahbar, Martin Medrano, Franck Diaz‐Garelli, Cosme Gonzalez Villaman, Sepideh Saroukhani, Sori Kim, Amirali Tahanan, Yahaira Franco, Gelanys Castro‐Tejada, Sarah A. Diaz, Manouchehr Hessabi, Sean I. Savitz

**Affiliations:** ^1^ Institute for Stroke and Cerebrovascular Disease The University of Texas Health Science Center at Houston Houston Texas USA; ^2^ Biostatistics/Epidemiology/Research Design (BERD) Core, Center for Clinical and Translational Sciences (CCTS) The University of Texas Health Science Center at Houston Houston Texas USA; ^3^ Department of Epidemiology, Human Genetics, and Environmental Sciences, School of Public Health The University of Texas Health Science Center at Houston Houston Texas USA; ^4^ Division of Clinical and Translational Sciences, Department of Internal Medicine, McGovern Medical School The University of Texas Health Science Center at Houston Houston Texas USA; ^5^ Department of Medicine, School of Medicine Pontificia Universidad Catalica Madre and Maestra (PUCMM) Santiago Dominican Republic; ^6^ Department of Public Health Sciences, College of Health and Human Services University of North Carolina at Charlotte Charlotte North Carolina USA; ^7^ Department of Neurosurgery Clínica Corominas Santiago Dominican Republic; ^8^ Department of Biostatistics & Data Science, School of Public Health The University of Texas Health Science Center at Houston Houston Texas USA; ^9^ Department of Neurology Clínica Corominas Santiago Dominican Republic; ^10^ Department of Neurology, McGovern Medical School The University of Texas Health Science Center at Houston Houston Texas USA

## Abstract

Stroke is the second leading cause of mortality globally with higher burden and younger age in low‐middle income countries (LMICs) than high‐income countries (HICs). However, it is unclear to what extent differences in healthcare access and quality (HAQ) and prevalence of risk factors between LMICs and HICs contribute to younger age of stroke in LMICs. In this systematic review, we conducted meta‐analysis of 67 articles and compared the mean age of stroke between LMICs and HICs, before and after adjusting for HAQ index. We also compared the prevalence of main stroke risk factors between HICs and LMICs. The unadjusted mean age of stroke in LMICs was significantly lower than HICs (63.1 vs. 68.6), regardless of gender (63.9 vs. 66.6 among men, and 65.6 vs. 70.7 among women) and whether data were collected in population‐ (64.7 vs. 69.5) or hospital‐based (62.6 vs. 65.9) studies (all *p* < 0.01). However, after adjusting for HAQ index, the difference in the mean age of stroke between LMICs and HICs was not significant (*p* ≥ 0.10), except among women (*p* = 0.048). In addition, while the median prevalence of hypertension in LMICs was 23.4% higher than HICs, the prevalence of all other risk factors was lower in LMICs than HICs. Our findings suggest a much larger contribution of HAQ to the younger mean age of stroke in LMICs, as compared with other potential factors. Additional studies on stroke care quality and accessibility are needed in LMICs.

## Introduction

Stroke is the second leading cause of morbidity and mortality,^1^, ^2^ and the third leading cause of disability^3^, ^4^ globally. The burden of stroke in low‐middle income countries (LMICs) is higher than in high‐income countries (HICs) and this gap is increasing.^4^, ^5^ Recent global burden of disease (GBD) studies reported that although stroke incidence, prevalence, and mortality rates decreased in HICs in the recent decades, there have been no significant changes in the incidence of stroke in LMICs.^6^ One of the reasons for this difference is limited access to diagnostic techniques for identifying stroke subtypes in LMICs, as CT imaging or MRI facilities are not readily available or not affordable by some LMICs.^2^ However, it is not clear how much of these differences could be attributed to differences in access to diagnostic techniques. Also, evidence shows that the age‐adjusted incidence of stroke in LMICs exceeded that in HICs by 20% for the first time during 2000–2008.^7^ Despite a global decline in the age‐standardized stroke death rate, the decline has been slower in LMICs than HICs.^8^ Specifically, the mortality rate of stroke has been reduced to half in HICs but only reduced by 15% in LMICs.^9^ Furthermore, the global absolute number of people who developed new stroke, died, survived, or remained disabled from stroke has almost doubled from 1990 to 2017 with a major bulk of stroke burden in LMICs in 2017 (80% all incident strokes, 77% all stroke survivors, and 87% of all deaths from stroke).^6^, ^10^, ^11^

Although aging is postulated as an explanation for the increased rates of stroke over time, about one‐fourth of ischemic strokes occur in working‐aged individuals in the HICs, and new strokes strike an estimated 3.6 million young people each year.^12^ There is also evidence suggesting that stroke may occur at a younger age in LMICs as compared to HICs.^13^, ^14^, ^15^, ^16^ However, despite over two‐thirds of stroke deaths worldwide occurring in LMICs, high quality and reliable data from population‐based prospective studies are sparse, especially in LMICs.^17^ For example, the data on stroke epidemiology and mortality may be hospital‐based in many LMICs, potentially ignoring non‐hospitalized cases and lacking generalizability to the whole population.^18^ In addition, limited number of studies from LMICs provide reliable data on the age of stroke and its differences by sex, stroke etiology, and subtypes.

The increasing prevalence of known risk factors such as high blood pressure, dyslipidemia, diabetes, obesity, and atrial fibrillation (AF) are possible reasons driving stroke rates and stroke burden.^19^, ^20^, ^21^ Although global data show that 90.5% of the stroke burden are attributable to such modifiable risk factors,^22^ the role of differences in their epidemiology along with other stroke risk factors between HICs and LMICs and their possible contribution to the increasing burden and younger age of stroke in LMICs is unclear. Therefore, in addition to comparing epidemiology of the aforementioned risk factors, we also sought to determine if other factors contribute to the younger age of stroke in LMICs. For example, studies have shown differences in access to healthcare, health technologies, and relative rates of stroke risk factors between HICs and LMICs.^22^ A systematic analysis suggested that healthcare access and quality (HAQ) may have direct impacts on health outcomes, including stroke mortality.^11^, ^23^, ^24^, ^25^ However, to our knowledge, the possible contribution of HAQ to the younger age of stroke in LMICs is unclear.

We systematically review the existing literature on the mean age of stroke in different countries and compare it between HICs and LMICs by gender, stroke subtypes, and whether the data were collected in a population‐ or hospital‐based setting, before and after adjusting for HAQ index. In addition, using publicly available global data, we compared the prevalence of main stroke risk factors including hypertension, diabetes, high cholesterol, obesity, and AF between HICs and LMICs.

## Methods

### Economic classification

We used publicly available data from the World Bank's 2020 fiscal year (2020–2021) classification of countries' income levels based on gross national income (GNI) per capita (current US$) of the previous calendar year (2019): low‐income (≤$1035 GNI), lower‐middle income ($1036 to $4045 GNI), upper‐middle income ($4046 to $12,535 GNI), and high‐income (≥$12,536 GNI).^26^ In this study, we have classified countries with low‐, lower‐middle‐, and upper‐middle income economies as LMICs.

### Mean age of stroke in LMICs and HICs

#### Study design, literature search strategy, and data extraction

This is a systematic review of peer‐reviewed articles to assess the differences in the mean age of stroke in HICs and LMICs. Following the Preferred Reporting Items for Systematic Reviews and Meta‐Analyses (PRISMA) guidelines,^27^ (PRISMA Checklist is in Table S6) we initially searched PubMed database using MeSH terms through a concept‐based search strategy with keywords “stroke” AND “mean age” to identify articles. Then, we restricted the searches to articles that were published in English from January 2000 to July 2021 with human adult patients. Duplicates were removed and the titles of the remaining articles were reviewed to exclude articles with irrelevant titles. We further reviewed the abstracts, and full texts of the remaining articles to identify the list of articles that met eligibility criteria to be included in meta‐analysis. We extracted data from each article for the mean age of stroke by gender, stroke subtypes, and whether the data were collected in a hospital‐ or population‐based setting and organized the information into a Microsoft Excel database. The database was organized according to the author, publication year, country name, region, population data, and economic classification as LMICs or HICs.

### HAQ index

We used publicly available GBD^28^ data on HAQ index, which was constructed using GBD 2016 estimates for 32 causes of death that may be treatable with effective care to approximate personal HAQ for countries and territories, as well as selected subnational locations, over time.^23^ In brief, GBD 2016 Healthcare Access and Quality Collaborators used estimates for age‐ and risk‐standardized death rates from 24 non‐cancer causes and age‐standardized mortality‐to‐incidence ratios for eight cancers that are considered amenable to healthcare, to construct the HAQ Index providing an overall score of 0–100 (0 and 100 indicate worst and best, respectively).^23^ For our systematic review, we only analyzed the country‐level HAQ index, which accounted for 195 countries.

### Meta‐analysis

As variances for the variables in each country were not available, we excluded the estimation of variance for each country and focused on estimation of mean age for these variables. Based on mean estimation from each country, we have estimated the weighted sum and variance, using on the population size in 2019 by World Bank as weights. We used univariable weighted generalized linear models (GLMs) to compare the mean age of stroke (i.e., dependent variable) between LMICs and HICs, as well as for subgroup analyses comparing the mean age of stroke between LMICs and HICs by gender, stroke subtypes, and whether the data sources were population‐based or hospital‐based. In addition, we used multivariable GLMs that account for population weights to compare the mean age of stroke between LMICs and HICs, as well as subgroup analyses adjusted for HAQ index. As variance data were not available within the country level, the test for homogeneity of variance was not performed.

### Prevalence of stroke risk factors

#### Data source identification and definition of risk factors

We focused on five stroke risk factors including hypertension, diabetes, high cholesterol, obesity, and AF with the most recent version of publicly available global country‐level data observatories from either World Health Organization (WHO),^29^ GBD,^28^ or International Diabetes Foundation (IDF).^30^ Specifically, we used 2015 WHO data^31^ for prevalence of hypertension (systolic blood pressure ≥140 or diastolic blood pressure ≥90), 2008 WHO data^32^ for prevalence of high cholesterol (total cholesterol ≥6.2 mmol/L), 2016 WHO data^33^ for prevalence of overweight and obesity (body mass index [BMI] ≥25 and BMI ≥30, respectively), and 2019 GBD data^28^ for prevalence of AF. For prevalence of diabetes (fasting blood glucose ≥7.0 mmol/L or on medication), we identified data from 2019 GBD,^28^ 2014 WHO,^34^ to 2019 IDF.^30^ For all the aforementioned risk factors, we only included country‐level data.

#### Data extraction and analysis

Data on the prevalence of stroke risk factors from different resources were standardized to percentage (%). Available datasets for each stroke risk factors were all downloaded in the form of Microsoft Excel files, and were merged by data source, publication year, country name, region, population data, economic classification, LMICs, and HICs binary data based on the economic classification, age range (if available), gender (if available), prevalence percentage, and its range (minimum, maximum). Since the country‐level variance data for the prevalence of risk factors were not available, we excluded estimation of variance for each country.

All statistical procedures were conducted using SAS 9.4 software.^35^ All hypotheses were tested at a 5% level of significance.

## Results

**Figure 1:**
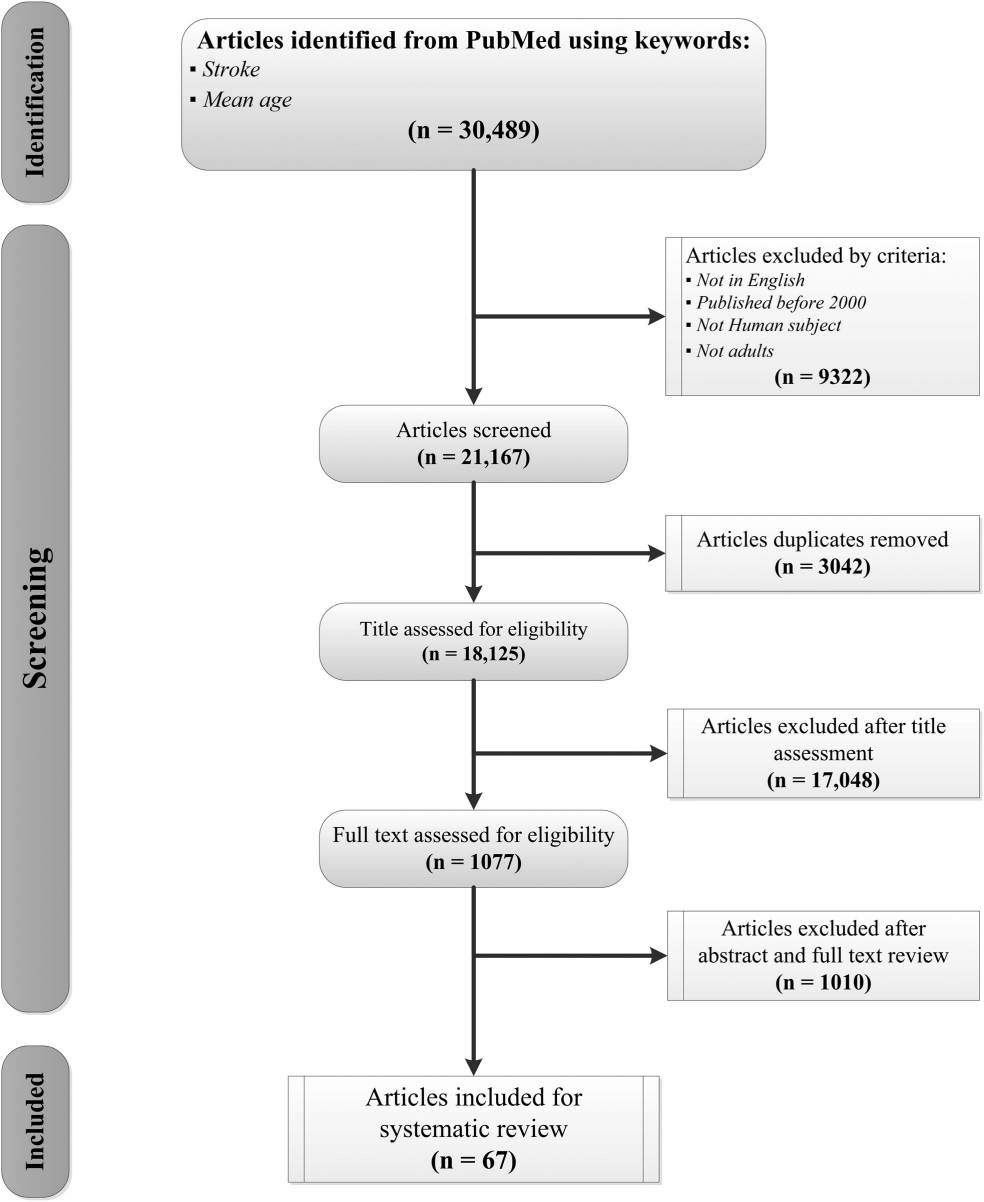
Systematic review flow chart following PRISMA guideline.

We identified 30,489 peer‐reviewed articles in our initial PubMed search. We only included articles in English, with adult human subjects that were published after 2000 (21,167 articles). We also removed 3042 duplicate articles before conducting other eligibility assessments. The remaining 18,125 articles were further narrowed down to 1077 by reviewing the titles. After reviewing the abstracts and full text of the 1077 articles, 67 articles that reported age of stroke in adult populations met the inclusion criteria (Fig. 1).^21^, ^29^, ^31^, ^32^, ^33^, ^34^, ^36^, ^37^, ^38^, ^39^, ^40^, ^41^, ^42^, ^43^, ^44^, ^45^, ^46^, ^47^, ^48^, ^49^, ^50^, ^51^, ^52^, ^53^, ^54^, ^55^, ^56^, ^57^, ^58^, ^59^, ^60^, ^61^, ^62^, ^63^, ^64^, ^65^, ^66^, ^67^, ^68^, ^69^, ^70^, ^71^, ^72^, ^73^, ^74^, ^75^, ^76^, ^77^, ^78^, ^79^, ^80^, ^81^, ^82^, ^83^, ^84^, ^85^, ^86^, ^87^, ^88^, ^89^, ^90^, ^91^, ^92^, ^93^, ^94^, ^95^

**Figure 2:**
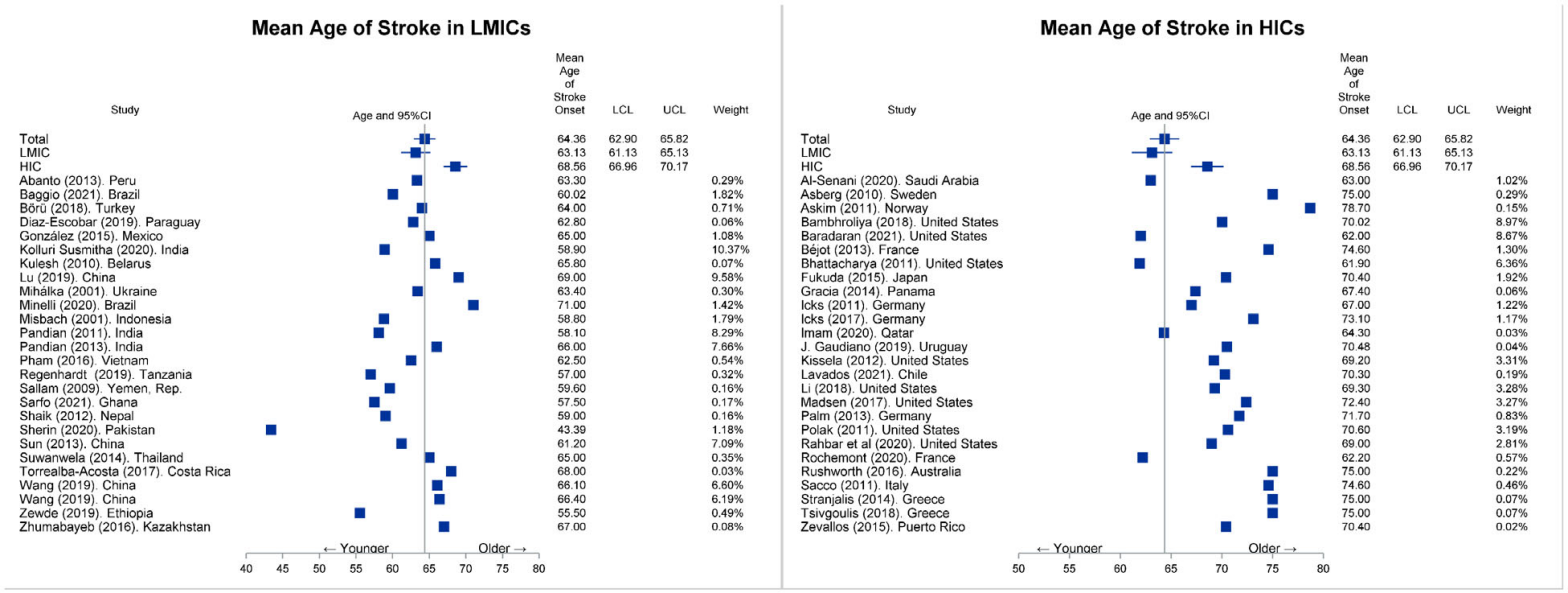
Forest plot on mean age of stroke in LMICs and HICs. LMICs, low‐middle income countries; HICs, high‐income countries. [Colour figure can be viewed at wileyonlinelibrary.com]

As shown in Table 1, the mean age of stroke in all countries was 64.4 years (95% CI: [62.9, 65.8]), and patients with stroke in LMICs were significantly younger than those in HICs (63.1 years vs. 68.6 years, *p* < 0.01). However, after adjusting for HAQ index, mean age difference of stroke between LMICs and HICs was not statistically significant (adjusted mean age difference [LMICs mean−HICs mean] [95% CI]: −0.9 [−4.2, 2.5], *p* = 0.61). The subgroup analysis with gender‐specific data revealed that the overall mean age of stroke was 64.1 years among men and 66.1 years among women. In the unadjusted model, the mean age of stroke patients in LMICs were significantly lower than that in HICs among both men and women, with larger difference in women (unadjusted mean age difference [95% CI]: females −5.1 years [−9.5, −0.8] vs. men −2.8 years [−7.1, 1.6]). However, only the mean age difference of female patients remained significant after adjusting for HAQ index (adjusted mean age difference [95% CI]: women −6.8 years [−13.5, −0.2], *p* = 0.048 vs. men −1.5 years [−6.1, 3.0], *p* = 0.49). Based on stroke subtypes, mean age of stroke was 67.5 years for ischemic stroke and 61.6 years for intracerebral hemorrhage. While the unadjusted mean age of ischemic stroke in LMICs was significantly lower than that in HICs (66.6 years vs. 69.9 years, *p* = 0.03), the difference was not statistically significant after accounting for HAQ index (66.6 years vs. 66.5 years, *p* = 0.96). Although the unadjusted mean age of patients with intracerebral hemorrhage in LMICs were younger than that in HICs, the difference was not statistically significant (60.9 years vs. 73.2 years, *p* = 0.46). However, mean age difference of patients with intracerebral hemorrhage between LMICs and HICs became marginally significant after adjusting for HAQ index (adjusted mean age difference [95% CI]: −9.5 years [−21.7, 2.6], *p* = 0.10). Similarly, overall mean age of patients with stroke was 66.3 years based on population‐based studies, and 62.9 years based on hospital‐based studies. Patients with stroke in LMICs were significantly younger than those in HICs, regardless of whether the data were from population‐ or hospital‐based studies, though with a larger difference observed in population‐based studies (unadjusted mean age difference between LMICs and HICs [95% CI]: population‐based studies −4.8 years [−8.8, −0.8] vs. hospital‐based studies −3.3 years [−6.7, 0.0]). However, such differences were not significant after adjusting for HAQ index (adjusted mean age of patients in LMICs vs. HICs: hospital‐based studies 63.9 years vs. 62.3 years, *p* = 0.51, population‐based studies 67.1 years vs. 65.8 years, *p* = 0.56). Details on the comparison of mean age of patients with stroke among LMICs and HICs are displayed in Table 1 and Figure 2.

**Table 1:** Meta‐analysis on mean age of stroke in LMICs and HICs, before and after adjusting for HAQ index.

Mean age of stroke (years)	Unadjusted					Adjusted^1^			
	All	LMICs	HICs	Mean difference^2^	*p* value^3^	LMICs	HICs	Mean difference^2^	*p* value^3^
Overall (*N* = 52)^4^	64.4 (62.9, 65.8)	63.1 (61.1, 65.1)	68.6 (66.9, 70.2)	−5.4 (−7.9, −2.9)	< 0.01	65.1 (63.6, 66.6)	65.9 (63.3, 68.5)	−0.9 (−4.2, 2.5)	0.61
By gender^5^									
Male (*N* = 19)	64.1 (62.8, 65.4)	63.9 (62.0, 65.7)	66.6 (63.6, 69.7)	−2.8 (−7.1, 1.6)	< 0.01	64.5 (62.9, 66.0)	66.0 (61.9, 70.1)	−1.5 (−6.1, 3.0)	0.49
Female (*N* = 19)	66.1 (64.2, 68.0)	65.6 (62.8, 68.3)	70.7 (67.0, 74.5)	−5.1 (−9.5, −0.8)	< 0.01	64.8 (62.6, 67.0)	71.6 (65.6, 77.6)	−6.8 (−13.5, −0.2)	0.048
By stroke sub‐type									
Ischemic (*N* = 16)^6^	67.5 (66.1, 69.0)	66.6 (65.2, 68.1)	69.9 (67.1, 72.8)	−3.3 (−6.1, −0.6)	0.03	66.6 (65.6, 67.5)	66.5 (64.4, 68.6)	0.1 (−2.3, 2.4)	0.96
Intracerebral hemorrhage (*N* = 7)^7^	61.6 (57.9, 65.3)	60.9 (58.5, 63.4)	73.2 (45.2, 101.3)	−12.3 (−25.1, 0.4)	0.46	59.9 (56.8, 62.9)	69.4 (56.8, 81.9)	−9.5 (−21.7, 2.6)	0.10
By method of data collection									
Hospital‐based (*N* = 26)^8^	62.9 (61.3, 64.5)	62.6 (60.5, 64.7)	65.9 (63.0, 68.8)	−3.3 (−6.7, 0.0)	< 0.01	63.9 (62.4, 65.3)	62.3 (58.1, 66.6)	1.5 (−3.2, 6.3)	0.51
Population‐based (*N* = 28)^9^	66.3 (64.1, 68.4)	64.7 (61.0, 68.4)	69.5 (67.6, 71.4)	−4.8 (−8.8, −0.8)	< 0.01	67.1 (64.8, 69.3)	65.8 (62.5, 69.1)	1.3 (−3.2, 5.9)	0.56

Numbers are displayed as mean (95% CI), unless otherwise noted. LMICs, low‐middle income countries; HICs, high‐income countries; HAQ, health access and quality.

^1^ Adjusted for HAQ index.

^2^ Mean difference = pooled mean age difference (LMICs−HICs).

^3^ Pooled/Satterthwaite for variance estimation for *t*‐test.

^4^ LMICs: *N* = 26, HICs *N* = 26.

^5^ LMICs: *N* = 9, HICs *N* = 10.

^6^ LMICs: *N* = 9, HICs *N* = 7.

^7^ LMICs: *N* = 4, HICs *N* = 3.

^8^ LMICs: *N* = 16, HICs *N* = 10.

^9^ LMICs: *N* = 12, HICs *N* = 16.

In Table 2, we compared median and range of prevalence of the five main known risk factors for stroke including hypertension, diabetes, high cholesterol, obesity, and AF, between LMICs and HICs using publicly available data. Our findings show that median prevalence of hypertension was 23.4% higher in LMICs than HICs (25.8% vs. 20.9%, difference ratio [DR] = 23.4%). However, median prevalence of all other risk factors was lower in LMICs as compared to HICs (Table 2). For diabetes, the median prevalence of both type I and II diabetes in LMICs was 3.4% higher than that in HICs (median prevalence: 7.2% vs. 7.0%, DR = 3.4%). However, specific median prevalence of type I and type II diabetes was 48.9% and 10.4% lower in LMICs than HICs (DR = −18.6%), respectively (median prevalence in LMICs vs. HICs: type I diabetes 0.2% vs. 0.4%, and type II diabetes 5.5% vs. 6.1%). High cholesterol was 57.0% less prevalent in LMICs compared to HICs, which showed the largest relative difference among the five risk factors (7.3% vs. 16.9%, DR = −57.0%).

**Table 2:** Comparison of the prevalence of stroke risk factors between LMICs and HICs.

Median estimated prevalence (%)	Worldwide	LMICs	HICs	Difference ratio (%)^1^
Hypertension^2^	24.6 (11, 33.4)	25.8 (13.7, 33.4)	20.9 (11.0, 32.4)	23.4
Diabetes^3^				
All	7.2 (1.0, 30.5)	7.2 (1.0, 30.5)	7.0 (2.1, 29.2)	3.4
Type I	0.2 (0.1, 0.9)	0.2 (0.1, 0.5)	0.4 (0.1, 0.9)	−48.9
Type II	5.7 (2.3, 20.9)	5.5 (2.3, 20.9)	6.1 (2.7, 17.4)	−10.4
High cholesterol^4^	9.1 (2.5, 29.1)	7.3 (2.5, 15.7)	16.9 (8.7, 29.1)	−57.0
Obesity^5^				
BMI ≥25 (overweight)	54.9 (18.3, 88.5)	48.1 (18.3, 83.5)	59.1 (27.2, 88.5)	−18.6
BMI ≥30 (obese)	20.6 (2.1, 61)	17.1 (2.1, 52.9)	23.1 (4.3, 61.0)	−26.0
Atrial fibrillation^6^	0.55 (0.16, 1.44)	0.48 (0.16, 1.18)	0.87 (0.33, 1.44)	−45.0

Numbers are displayed as median (minimum, maximum), unless otherwise noted. LMICs, low‐middle income countries; HICs, high‐income countries.

^1^ Difference ratio (%) = (LMICs median−HICs median)/HICs median × 100.

^2^ 2015 World Health Organization (WHO)^31^ data from 133 LMICs and 60 HICs.

^3^ 2019 Global Burden of Disease (GBD),^28^ 2014 WHO,^86^ and 2019 International Diabetes Foundation^30^ data from 134 LMICs and 66 HICs.

^4^ 2008 WHO^32^ data from 131 LMICs and 60 HICs.

^5^ 2016 WHO^33^ data from 133 LMICs and 60 HICs.

^6^ 2019 GBD^28^ data from 134 LMICs and 66 HICs.

Similarly, the median prevalence of obesity and AF were 26.0% and 45.0% lower in LMICs than HICs, respectively (DR = −45.0%). Additional detailed data on comparisons of age‐ and gender‐specific prevalence of each risk factor between LMICs and HICs are provided in Tables S1–S1.

## Discussion

In this systematic review, we summarized the relevant published literature on the mean age of stroke in LMICs and HICs. While the overall mean age of stroke among all countries was 64.4 years, patients with stroke in LMICs were about 5.5 years younger than those in HICs. Moreover, the mean age of stroke patients in LMICs was significantly younger than in HICs regardless of the patients' gender, stroke sub‐types, and whether the data were collected at population or hospital level. However, after adjusting for HAQ index, the difference in the mean age of stroke between LMICs and HICs did not remain significant in any main and subgroup analyses, except among women, where adjusted mean age of stroke patients remained significantly younger in LMICs than HICs. In addition, we found that hypertension was the most prevalent risk factor in LMICs, with a median prevalence that was 23.4% higher than that in HICs. However, median prevalence of all other risk factors was lower in LMICs compared to HICs.

Our findings on the younger age of stroke in LMICs than HICs are consistent with findings from a review of 23 population‐based studies indicating a moderate positive correlation (*r* = 0.60) between country's SES measures including lower per capita gross domestic product (GDP) and total health expenditures per capita, and occurrence of stroke at a younger age.^96^ However, they did not compare mean age at stroke between countries with low and high GDP or conduct a meta‐analysis to report a pooled estimate. Our findings are also consistent with the GBD data showing that patients with incident and fatal ischemic and hemorrhagic stroke in LMICs were 3–5 years younger than those in HICs. Their findings suggested that while the worldwide mean age of people with incident and fatal ischemic and hemorrhagic stroke has increased from 1990 to 2017, this increase was most dramatically noted in HICs.^6^, ^16^ However, they did not report the mean age of stroke by gender subgroups or adjust for any potential confounders. A recent study, “Global Outcome Assessment Lifelong after stroke in young adults” (GOAL) initiative, combined data from individual young patients with stroke from 32 cohorts in 29 countries and reported that LMICs had less vascular risk factors, but a higher 3‐month mortality than those from HICs (OR = 2.49; 95% CI 1.42–4.36).^5^

To our knowledge, our study is the first to report that the significant difference in the mean age of stroke between LMICs and HICs diminished after accounting for health care access and quality as measured by country‐level HAQ index. This finding suggests that the younger age of stroke in LMICs is not necessarily related to the overall country income level, and that the HAQ differences between LMICs and HICs could explain, at least in part, the higher burden due to younger age of stroke in LMICs.

Despite the decreasing trends in the incidence and mortality of new and recurrent stroke especially in HICs, a substantial portion of strokes in both HICs and LMICs are further preventable by proper management of major modifiable risk factors for stroke such as hypertension, diabetes, hyperlipidemia, obesity, and antithrombotic therapy.^97^ For example, despite the fact that hypertension is the most common modifiable risk factor for stroke, affecting about one‐third of US adults over 20 years of age, blood pressure was controlled in only about half of adults in the US with hypertension.^98^ Findings of the cross‐sectional phase of the Prospective Urban Rural Epidemiology (PURE) study also showed a large gap between detection and control of hypertension across all countries. Specifically, they have shown that awareness, treatment, and control of hypertension were lower in LMICs than HICs, as well as in rural than urban settings within LMICs.^99^ For example, a survey of prevention strategies in Pakistan revealed that only 63% of physicians prescribed antihypertensive therapy if blood pressure was 140/90 mmHg or greater, and 75% did not routinely check the lipid profile.^4^ This lack of awareness, under‐diagnosis and suboptimal management of modifiable risk factors can also contribute to the high burden and younger age of stroke in LMICs despite the overall lower prevalence of other stroke risk factors that we observed in our analyses.

Another possible explanation for the younger age of stroke in LMICs may be the limited availability of accurate data on the prevalence of risk factors and stroke, mainly due to lack of infrastructure for high‐quality prospective, population‐based, and clinical studies in LMICs. The World Stroke Organization‐WHO‐Lancet Neurology Commission on Stroke has recently published the results of a survey assessing the status of stroke services (surveillance, prevention, acute stroke, and rehabilitation) in LMICs compared to HICs. Their findings showed that only a small proportion of LMICs has stroke surveillance activities that is stroke registries, active or recently completed risk factors surveys, participation in either population‐based or clinical research for stroke, etc.^89^ This lack of prospective studies and surveillance result in data that significantly underestimate the actual prevalence of stroke and cardiovascular risk factors in the LMICs as compared to HICs. For example, Miranda et al^100^ have shown that data on the distribution of cardiovascular risk factors in Latin American and Caribbean (LAC) region are not only very limited, but also the available data have significant variation in the levels of prevalence. For instance, the CARMELA study^101^ that was conducted in seven major urban cities in LAC, reported markedly different hypertension prevalence ranging from 24% to 29% in Buenos Aires (Argentina), Santiago (Chile), and Barquisimeto (Venezuela), whereas from 9% to 13% in Quito (Ecuador), Mexico City (Mexico), Lima (Peru), and Bogota (Colombia). However, the prevalence of diabetes in these cities were similar to global estimates that was about 7%.^101^ Therefore, developing capacity for high quality, population‐based, and clinical research in LMICs is warranted.

Along with the aforementioned key contributors to the younger age of stroke in LMICs, there are other potential risks, such as household air pollution, urbanization, poor diet, and social vulnerability that vary by country's economic level.^102^, ^103^ For example, there is evidence suggesting 21% of all stroke deaths are attributable to air pollution globally,^104^ and 87% of this burden occurs in LMICs, particularly concentrated in sub‐Saharan Africa and South and East Asia.^105^ However, reliable global data about these factors are not available, especially from LMICs. Therefore, burden of these risk factors and their possible associations with stroke, especially in LMICs, require further investigation.

### Limitations

We acknowledge certain limitations of our study. First, although we made every attempt to include as much data as possible from LMICs, such data particularly from population‐based studies with high methodological quality are scarce and publication bias is also possible. Second, LMICs might have had lower rates of neuroimaging investigations than HICs, which can limit the ability to distinguish between stroke types particularly in LMICs. Furthermore, for the stroke risk factors, we only considered main stroke risk factors with available data covering most of the HICs and LMICs. There are additional risk factors for stroke such as smoking, level of physical activity, diet/nutrition, and air pollution that are not included in this analysis. Since the country‐level variance data were not available for the age of stroke and the prevalence of risk factors, homogeneity of variance test was not performed.

## Conclusions

In our systematic review, we found that although strokes occur at younger ages in LMICs as compared to HICs regardless of gender, subtype of stroke and whether the data are collected at population or hospital level, the younger age of stroke in LMICs than in HICs is not necessarily related to the overall country income level. Specifically, our analyses suggest health care access and quality as a main contributor to higher burden and younger age of stroke in all countries. In addition, women in LMICs may be more susceptible to stroke at younger ages than those in HICs even after accounting for HAQ. Therefore, improving the quality and accessibility of care, especially for vulnerable populations such as women can reduce the gap in mean age and burden of stroke between LMICs and HICs. In addition, developing capacity for high quality population‐based and clinical research in LMICs is crucial.

## Conflict of Interest

The authors declare that they have no conflict of interest.

## Supporting information


**Table S1.** Comparison of the prevalence of hypertension (systolic blood pressure ≥140 or diastolic blood pressure ≥90) among adults from age group of ≥25 years between LMICs and HICs.
**Table S2.** Comparison of the prevalence of diabetes (fasting blood glucose ≥7.0 mmol/L or on medication) between LMICs and HICs.
**Table S3.** Comparison of the prevalence of high cholesterol (total cholesterol ≥6.2 mmol/L) among adults from age group of ≥25 years between LMICs and HICs.
**Table S4.** Comparison of the prevalence of obesity (BMI ≥30) and overweight (BMI ≥25) between LMICs and HICs.
**Table S5.** Comparison of the prevalence of atrial fibrillation between LMICs and HICs.
**Table S6.** PRISMA 2020 Checklist.
